# Analysis of nonlinear bending behavior of nano-switches considering surface effects

**DOI:** 10.1186/s11671-024-04030-8

**Published:** 2024-05-20

**Authors:** Fan Yang, Xuyang Wang, Xianlai Song, Weilin Yang

**Affiliations:** 1 Department of Engineering Mechanics, Faculty of Sciences, Xi’an University of Science and Technology, Xi’an, 710600 China; 2 Department of Mechanics, Faculty of Engineering, University of Alberta, Edmonton, AB T6G1H9 Canada

**Keywords:** Nano-switch, Surface effects, Nonlinear bending, Casimir force

## Abstract

Nano-switch structures are important control elements in nanoelectromechanical systems and have potential applications in future nanodevices. This paper analyzes the effects of surface effects, geometric nonlinearity, electrostatic forces, and intermolecular forces on the nonlinear bending behavior and adhesion stability of nano-switches. Based on the Von Karman geometric nonlinearity theory, four types of boundary conditions for the nano-switch structure were specifically calculated. The results show that surface effects have a significant impact on the nonlinear bending and adhesion stability of nano-switches. Surface effects increase the adhesion voltage of the nano-switch and decrease its adhesion displacement, and as the size of the nano-switch structure increases, the impact of surface effects decreases. A comparative analysis of the linear theory and the nonlinear theory results shows that the adhesion voltage predicted by the linear theory is smaller than that predicted by the nonlinear theory. The effect of geometric nonlinearity increases as the size of the nano-switch structure increases, as the distance between the electrodes increases, and as the aspect ratio of the nano-switch structure increases. These findings provide theoretical support and reference for the design and use of future nanodevices and nanoelectromechanical systems.

## Introduction

Nano-switches are important control elements in nanoelectromechanical systems. Compared with conventional semiconductor switches, nano-switch structures are simple, small in size, light in weight, and have a short response time. They are of great significance for achieving self-control, improving automation and intelligence levels, and enhancing system reliability and balance. Nano-switches are also an important component of the design of next-generation information storage, exchange, and logic circuits [1]. A nano-switch consists of a movable electrode and a fixed electrode. When an external voltage is applied between the two electrodes, the movable electrode undergoes bending deformation due to the electric field force generated by the applied voltage. As the voltage increases, the deformation of the movable electrode increases. When the voltage reaches a certain value, the movable electrode loses stability and adheres to the fixed electrode [2, 3].

Due to the nanoscale dimensions of the nano switch, the initial gap between the movable and fixed electrodes is only a few hundred nanometers or even tens of nanometers. At this scale, the molecular interaction forces between the two electrodes, such as Van der Waals force and Casimir force, cannot be ignored. When the electrode spacing is greater than 20 nm, the Casimir force dominates, while when the electrode spacing is less than 20 nm, the Van der Waals force dominates. In order to analyze the effect of molecular interaction forces on the adhesion characteristics of the nano switch, Ataei et al. [4] used the strain gradient theory to study the adhesion instability of a cantilevered nano actuator made of functionally graded materials under the influence of electrostatic and molecular interaction forces. Ramezani et al. [5, 6] used a distributed parameter model to study the effect of Casimir force on the adhesion parameters of a cantilevered nano switch, and investigated the adhesion parameters under three different conditions: no external voltage, no Casimir force, and both considered. Attia et al. [7] comprehensively studied the effect of various parameters such as material relaxation time, persistent modulus, material length scale parameter, Casimir force, Van der Waals force, initial gap, and beam length on the adhesion response of viscoelastic cantilever beams.

At the nanoscale, the ratio of surface atoms to internal atoms in a material increases. At this scale, the surface effect of the nano-switch cannot be ignored in its adhesion characteristics. Soroush et al. [8] developed a double-layer nano-switch model that combines surface energy and microstructure coupling effects based on surface elasticity theory and consistent coupling stress theory. Keivani et al. [9] used surface elasticity theory to study the static and dynamic adhesion behavior of nano-switches made of conductive cylindrical nanowires. Ma et al. [10] used the Euler–Bernoulli beam model to investigate the influence of surface effects (including residual surface stress and surface elasticity) on the adhesion instability of nanoelectromechanical systems (NEMS) electrostatic switches, taking into account the nonlinear effects generated by driving electrostatic forces and Casimir forces. Fu [11] proposed an improved continuum model for electrically-driven nanobeam by introducing surface elasticity and discussed the effect of surface energy on static and dynamic responses, adhesion voltage, and adhesion time.

Under complex working conditions, nano-switches often undergo geometrically nonlinear deformations, and the influence of large deformations cannot be ignored. Wang et al. [12] studied the instability of nano-switches under electrostatic forces and intermolecular forces based on a geometrically nonlinear Euler–Bernoulli beam theory that considers surface energy. Bhojawala et al. [13] studied the influence of Van der Waals force on the instability and free vibration characteristics of electrostatically driven fixed-end beams under axial forces, considering the effects of edge fields, nonlinearities caused by mid-plane tension, and Van der Waals forces. Dai et al. [14] studied NEMS and their instability and nonlinear dynamic responses, considering both geometric nonlinearities and inertial nonlinearities based on the theory of surface elasticity.

This article introduces surface effects through an equivalent elastic stiffness and surface energy model. Based on the Von Karman geometric nonlinear theory and the minimum potential energy principle, the nonlinear bending control equation and boundary conditions for the nano switch considering surface effects are derived. The Galerkin method is used to solve the control equation of the nano switch. The nonlinear bending and its adhesion stability of the nano switch are revealed, and the effects of surface effects, geometric dimensions, intermolecular forces, and electric field edge effects on the nonlinear bending and adhesion instability characteristics of the nano switch are discussed.

## Nano-switch basic equation

**Fig. 1:**
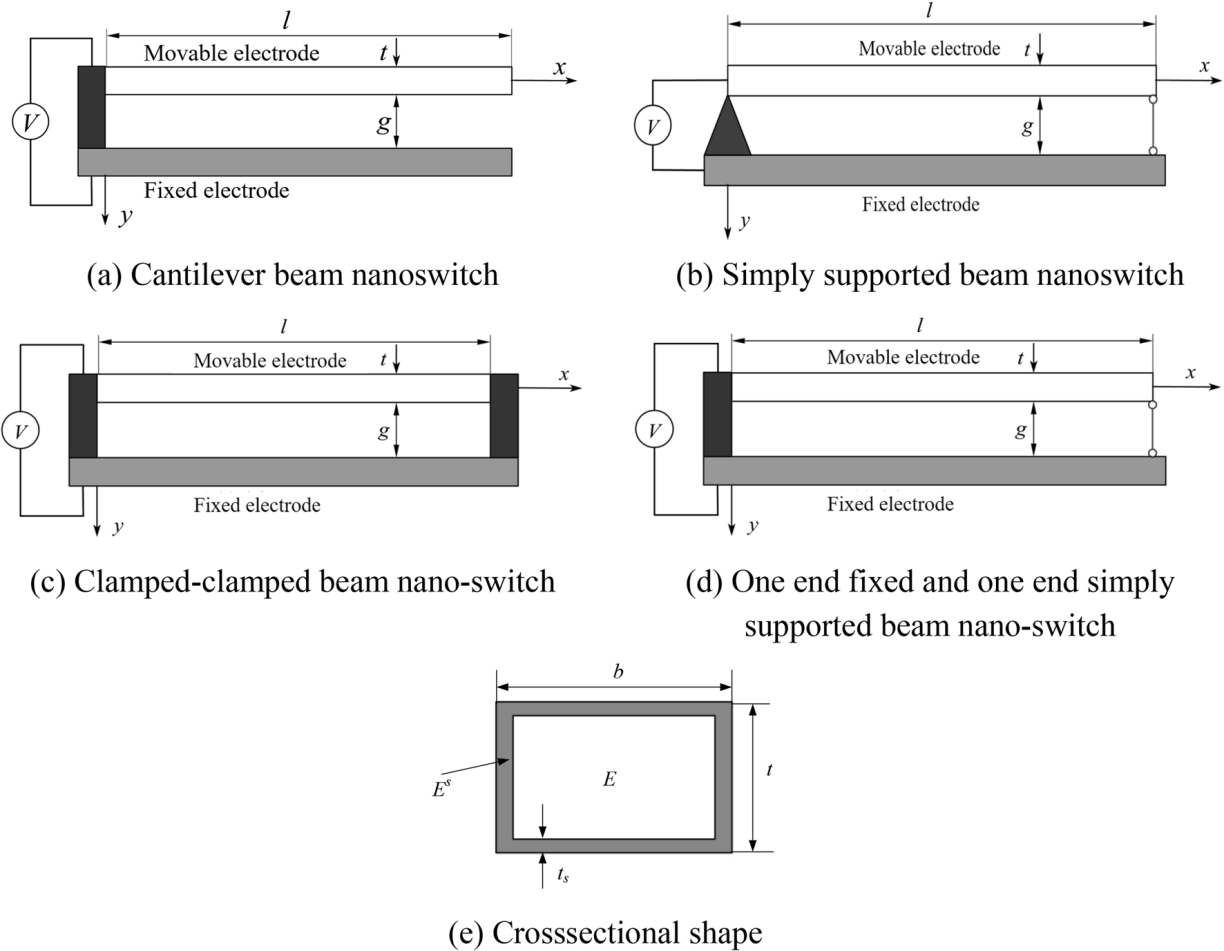
Nano-switch structure diagram and cross section shape

The schematic diagram of the nano-switch with different boundary conditions considering factors such as surface effects, Casimir force, and electric field force is shown in Fig. 1. In the model proposed in this paper, the nano-switch consists of a fixed electrode and a movable electrode. The length, width, and height of the movable electrode are denoted as *l*, *b*, and *t*, respectively, and the initial gap between the two plates is* g*. The coordinate system is established as shown in the figure, where the *x*-axis is established along the axis direction of the movable electrode and the *y*-axis is established perpendicular to the axis direction of the movable electrode. The displacement along the *x* and *y* axes is represented by *u*(*x*) and *w*(*x*), respectively, and both are functions of the spatial coordinate *x*.

In this paper's model, it is assumed that the initial distance between the two electrodes is greater than 20 nm, at which point the intermolecular force between the two electrodes is Casimir force [15].

1 $$F_{c} = \frac{{\pi^{2} \hbar cb}}{{240\left( {g - w} \right)^{4} }}$$

In the above equation, ℏ is the reduced Planck constant, which is equal to the Planck constant divided by 2π and has a value of 1.055 × 10^–34^ J; *c* is the speed of light, which has a value of 2.998 × 10^8^ ms^−1^.

**Fig. 2:**
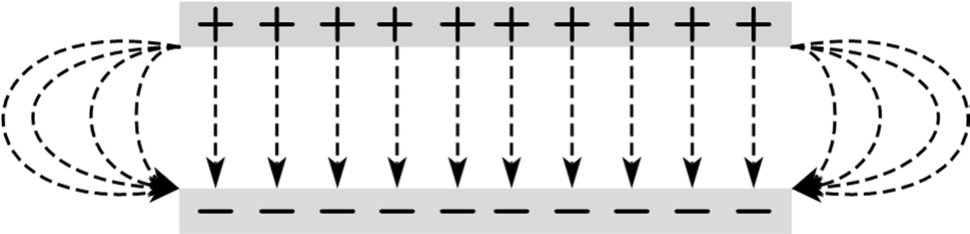
Electric fringing field effect diagram

When considering the electric field edge effect (Fig. 2), the electric field force between the two electrodes of the nano-switch can be expressed as [16, 17]:

2 $$F_{e} = \frac{{\varepsilon_{0} bV^{2} }}{{2\left( {g - w} \right)^{2} }}\left[ {1 + 0.65\frac{{\left( {g - w} \right)}}{b}} \right]$$

In the equation, *V* is the applied voltage, *ε*_0_ is the permittivity of vacuum, which has a value of 8.854 × 10^–12^ C^2^N^−1^ m^−2^.

The Casimir force and electric field force together constitute the sum of all distributed external forces acting on the movable electrode of the nano-switch.

3 $$F_{t} = F_{c} + F_{e}$$

In general, the distance between the two electrodes of the nano-switch is much smaller than the length of the movable electrode, so the mechanical behavior of the movable electrode is in a static elastic state. Therefore, we can analyze the mechanical behavior of the nano-switch using the Euler–Bernoulli beam model.

Due to the nanoscale size of the nano-switch, surface effects begin to emerge and affect the deformation and adhesion stability of the nano-switch. Therefore, the effective elastic modulus of the nano-switch considering surface effects should be determined by the elastic modulus and surface elastic modulus of the material. When the nano-switch undergoes bending deformation, the effective bending stiffness of the overall structure should be determined by the bending stiffness and surface bending stiffness of the nano-switch. In the model that considers large deformations, the effective tensile stiffness of the overall structure of the nano-switch should also be determined by the tensile stiffness and surface tensile stiffness of the nano-switch. Therefore, for a rectangular cross-section, the effective bending stiffness and effective tensile stiffness of the nano-switch structure considering surface effects can be expressed as [18, 19].

4 $$\left( {EI} \right)^{ * } = \frac{1}{12}Ebt^{3} + \frac{1}{2}E^{s} bt^{2} + \frac{1}{6}E^{s} t^{3}$$

5 $$\left( {EA} \right)^{ * } = Ebt + 2E^{s} \left( {b + t} \right)$$

In which, *E* is the elastic modulus of the beam body itself, and *E*^*S*^ is the surface elastic modulus.

The displacement component of the movable electrode can be expressed as:

6 $$u = u_{0} - y \cdot \frac{dw}{{dx}}, \, w = w_{0}$$

Here, *u*_*0*_ is the displacement component of the neutral axis in the *x*-direction, and *w*_*0*_ is the displacement component of the neutral axis in the *y*-direction.

According to the Von Karman geometric nonlinear strain hypothesis, the axial strain of the movable electrode can be expressed as:

7 $$\varepsilon_{x} = \frac{du}{{dx}} + \frac{1}{2}\left( {\frac{dw}{{dx}}} \right)^{2} - y \cdot \frac{{d^{2} w}}{{dx^{2} }}$$

The strain energy generated when the nano-switch bends is given by:

8 $$U_{b} = \int\limits_{V} {\int_{0}^{\varepsilon } {\sigma d\varepsilon dV} }$$

Taking the variation of the above equation yields

9 $$\delta U_{b} = \int_{0}^{l} {N\delta \left( {\frac{du}{{dx}}} \right)} dx + \int_{0}^{l} {N\frac{dw}{{dx}}\delta \left( {\frac{dw}{{dx}}} \right)} dx - \int_{0}^{l} {M\delta \left( {\frac{{d^{2} w}}{{dx^{2} }}} \right)} dx$$

in which

10 $$N = (EA)^{*} \left[ {\frac{du}{{dx}} + \frac{1}{2}\left( {\frac{dw}{{dx}}} \right)^{2} } \right], \, M = - (EI)^{*} \left( {\frac{{d^{2} w}}{{dx^{2} }}} \right)$$

Surface energy is the additional free energy generated by the free surface of a solid. When a solid deforms, new surface area is created and external work is required, and this energy should also be included in the total potential energy. The surface energy generated by a nano-switch during bending deformation is [20].

11 $$U_{s} = \gamma S_{0} + H\int_{0}^{l} {\left[ {1 + \frac{du}{{dx}} + \frac{1}{2}\left( {\frac{dw}{{dx}}} \right)^{2} } \right]dx}$$

where *γ* is the surface energy density; *S*_*0*_ is the initial surface area of the beam before deformation, which for a rectangular cross-section is 2(*b* + *t*)*l*; *H* is a coefficient related to the cross-section of the beam, which for a rectangular cross-section is 2*γ*(*b* + *t*).

Taking the variation of the above equation yields

12 $$\delta U_{s} = H\left[ {\delta u\left| {\begin{array}{*{20}c} l \\ 0 \\ \end{array} + \frac{dw}{{dx}}\delta w\left| {\begin{array}{*{20}c} l \\ 0 \\ \end{array} - \int_{0}^{l} {\frac{{d^{2} w}}{{dx^{2} }}\delta wdx} } \right.} \right.} \right]$$

The work done by the sum of distributed external forces on the movable electrode, which is composed of both the Casimir force and the electric field force is

13 $$W = \int_{0}^{l} {F_{t} } wdx$$

Taking the variation of the above equation yields.

Therefore, the total potential energy can be expressed as:

14 $$\Pi = U_{b} + U_{s} - W$$

Taking the variation of the above equation yields

15 $$\delta \Pi = \delta U_{b} + \delta U_{s} - \delta W$$

According to the principle of minimum potential energy, setting δΠ(*w*) = 0 and δΠ(*u*) = 0 leads to the governing equations of the nano-switch considering the surface effect.

16 $$\frac{dN}{{dx}} = 0$$

17 $$\frac{{d^{2} M}}{{dx^{2} }} + (N + H)\frac{{d^{2} w}}{{dx^{2} }} + \frac{dN}{{dx}} \cdot \frac{dw}{{dx}} + F_{t} = 0$$

And the overall boundary conditions.

18 $$\left( {N + H} \right)\delta u\left| {_{0}^{l} } \right. = 0$$

19 $$\left[ {\left( {N + H} \right)\frac{dw}{{dx}} + \frac{dM}{{dx}}} \right]\delta w\left| {_{0}^{l} = 0} \right.$$

20 $$M \cdot \delta \left( {\frac{dw}{{dx}}} \right)\left| {_{0}^{l} } \right. = 0$$

Integrating the first term of the control Eq. (16) over the length of the movable electrode l for twice and substituting the corresponding boundary conditions yields:

21 $$u = - \frac{1}{2}\int_{0}^{l} {\left( {\frac{dw}{{dx}}} \right)}^{2} dx + \frac{x}{2l}\int_{0}^{l} {\left( {\frac{dw}{{dx}}} \right)}^{2} dx$$

Substituting Eq. (21) into the second term of the control Eq. (17) results in a fourth-order nonlinear integro-differential control equation.

22 $$\left( {EI} \right)^{ * } \frac{{d^{4} w}}{{dx^{4} }} - H \cdot \frac{{d^{2} w}}{{dx^{2} }} - \frac{{\left( {EA} \right)^{ * } }}{2l}\int_{0}^{l} {\left( {\frac{dw}{{dx}}} \right)}^{2} dx \cdot \frac{{d^{2} w}}{{dx^{2} }} = F_{t}$$

To facilitate calculations, we introduce the non-dimensional variables *X* = *x*/*l* and *W* = *w*/*g*, and further non-dimensionalize the control equation, which considers the surface effect. The resulting equation is:

23 $$\frac{{d^{4} W}}{{dX^{4} }} - \eta \cdot \frac{{d^{2} W}}{{dX^{2} }} - \mu \cdot \int_{0}^{1} {\left( {\frac{dW}{{dX}}} \right)}^{2} dX \cdot \frac{{d^{2} W}}{{dX^{2} }} = \frac{\alpha }{{\left( {1 - W} \right)^{4} }} + \frac{{\beta^{2} }}{{\left( {1 - W} \right)^{2} }} + \frac{{\Upsilon \cdot \beta^{2} }}{{\left( {1 - W} \right)}}$$

where the definitions of the relevant parameters are as follows

24 $$\eta = \frac{{Hl^{2} }}{{\left( {EI} \right)^{ * } }},\mu = \frac{{g^{2} \left( {EA} \right)^{ * } }}{{2\left( {EI} \right)^{ * } }},\alpha = \frac{{\pi^{2} \overline{h}cbl^{4} }}{{240g^{5} (EI)^{*} }},\beta^{2} = \frac{{\varepsilon_{0} V^{2} bl^{4} }}{{2g^{3} (EI)^{*} }},\Upsilon = 0.65\frac{g}{b}$$

For a fixed-supported boundary nano-switch, its boundary conditions are as follows.

25 $$W = 0,\frac{dW}{{dX}} = 0$$

For simply supported boundaries

26 $$W = 0,\frac{{d^{2} W}}{{dX^{2} }} = 0$$

For free boundary

27 $$\frac{{d^{2} W}}{{dX^{2} }} = 0, \, \frac{{d^{3} W}}{{dX^{3} }} + \left[ {\eta + \mu \int_{0}^{1} {\left( {\frac{dW}{{dX}}} \right)^{2} dX} } \right] \cdot \frac{dW}{{dX}} = 0$$

## Solving method

We use the Galerkin method to separate variables and solve the nonlinear integral–differential control Eq. (23). According to the natural modes, we select the displacement function of the movable electrode of the nano-switch, i.e., $W\left( X \right) = \sum\limits_{i = 1}^{k} {C_{i} \varphi_{i} \left( X \right)}$, where k represents the degree of freedom of the movable electrode of the nanoswitch, Ci represents the displacement amplitude parameter, and *φ*_*i*_(*X*) represents the eigenfunction of the itch order of the movable electrode. This paper mainly considers the condition of contact instability of the nano-switch when it has a single degree of freedom (*k* = 1). The displacement function of the movable electrode of the nano-switch under single degree of freedom can be expressed as.

28 $$W\left( X \right) = C \cdot \varphi \left( X \right)$$

The first-order shape functions φ(X) for the movable electrode of the four different types of nano-switches with different boundary conditions are shown in Table 1.

**Table 1:** First-order shape functions φ(X) of the four different boundary conditions for the nano-switch

Boundary condition	First-order shape function *φ*(*X*)	latent root *λ*
Cantilever beams	$\varphi \left( X \right) = \cosh \lambda X - \cos \lambda X - \frac{\sinh \lambda - \sin \lambda }{{\cosh \lambda + \cos \lambda }}\left( {\sinh \lambda X - \sin \lambda X} \right)$	$\lambda = 1.8751$
Simply supported beams	$\varphi \left( X \right) = \sin \lambda X$	$\lambda = \pi$
Clamped–clamped beams	$\varphi \left( X \right) = \cosh \lambda X - \cos \lambda X - \frac{\cosh \lambda - \cos \lambda }{{\sinh \lambda - \sin \lambda }}\left( {\sinh \lambda X - \sin \lambda X} \right)$	$\lambda = 4.7300$
One end fixed and one end simply supported beam	$\varphi \left( X \right) = \cosh \lambda X - \cos \lambda X - \frac{\cosh \lambda + \cos \lambda }{{\sinh \lambda + \sin \lambda }}\left( {\sinh \lambda X - \sin \lambda X} \right)$	$\lambda = 3.9266$

By substituting Eq. (28) and the first-order shape functions *φ*(*X*) from Table 1 into the control Eq. (24), the Galerkin method is used for separation of variables. Multiplying each term in Eq. (23) by *φ*(*X*) and integrating over the domain *X* ∈ [0, 1] of the dimensionless nano-switch length, the equilibrium equation that only depends on the displacement parameter *C* can be obtained.

29 $$C \cdot \psi_{1} - C^{3} \cdot \psi_{2} - \beta^{2} \cdot \psi_{3} \left( C \right) + \psi_{4} \left( C \right) = 0$$

in which

30 $$\psi_{1} = \int_{0}^{1} {\left[ {\frac{{d^{4} \varphi \left( X \right)}}{{dX^{4} }} \cdot \varphi \left( X \right)} \right]} \, dX - \eta \int_{0}^{1} {\left[ {\frac{{d^{2} \varphi \left( X \right)}}{{dX^{2} }} \cdot \varphi \left( X \right)} \right]} \, dX$$

31 $$\psi_{2} = \mu \cdot \overline{S} \cdot \int_{0}^{1} {\left[ {\frac{{d^{2} \varphi \left( X \right)}}{{dX^{2} }} \cdot \varphi \left( X \right)} \right]} \, dX$$

32 $$\psi_{3} \left( C \right) = \int_{0}^{1} {\left[ {\frac{1}{{\left( {1 - C \cdot \varphi \left( X \right)} \right)^{2} }}} \right]} \, \varphi \left( X \right)dX - \int_{0}^{1} {\left[ {\frac{\Upsilon }{1 - C \cdot \varphi \left( X \right)}} \right]} \, \varphi \left( X \right)dX \,$$

33 $$\psi_{4} \left( C \right) = - \int_{0}^{1} {\left[ {\frac{\alpha }{{\left( {1 - C \cdot \varphi \left( X \right)} \right)^{4} }}} \right]} \, \varphi \left( X \right)dX$$

in which, $$\overline{S} = \int_{0}^{1} {\left[ {\frac{d\varphi \left( X \right)}{{dX}}} \right]}^{2} dX$$.

When the applied voltage reaches the pull-in voltage, the nano-switch becomes unstable and the movable electrode rapidly adheres to the fixed electrode. For a nano-switch in the pull-in instability state, its acceleration must be zero, so when taking the derivative of the left-hand side of Eq. (29) with respect to the unknown parameter *C*, it is also equal to zero, which means:

34 $$\psi_{1} - 3C^{2} \cdot \psi_{2} - \beta^{2} \cdot \frac{{d\psi_{3} \left( C \right)}}{dC} + \frac{{d\psi_{4} \left( C \right)}}{dC} = 0$$

By combining Eqs. (29) and (34) and eliminating the *β*^*2*^ term, the displacement undetermined parameter *C* in the stuck unstable state can be obtained, that is

35 $$\begin{gathered} C_{PI} \cdot \psi_{1} \cdot \frac{{d\psi_{3} \left( {C_{PI} } \right)}}{{dC_{PI} }} - \left( {C_{PI} } \right)^{3} \cdot \psi_{2} \cdot \frac{{d\psi_{3} \left( {C_{PI} } \right)}}{{dC_{PI} }} \hfill \\ + \psi_{4} \cdot \frac{{d\psi_{3} \left( {C_{PI} } \right)}}{{dC_{PI} }} - \psi_{1} \cdot \psi_{3} + 3\left( {C_{PI} } \right)^{2} \cdot \psi_{2} \cdot \psi_{3} - \frac{{d\psi_{4} \left( {C_{PI} } \right)}}{{dC_{PI} }} \cdot \psi_{3} = 0 \hfill \\ \end{gathered}$$

Solving Eq. (35) yields the value of the displacement undetermined parameter* C*_*PI*_ in the pull-in unstable state, and then the corresponding dimensionless pull-in voltage *β*_*2*_ value can be obtained by solving Eq. (29), namely,

36 $$\beta^{2} = \frac{{C \cdot \psi_{1} - C^{3} \cdot \psi_{2} + \psi_{4} \left( C \right)}}{{\psi_{3} \left( C \right)}}$$

Substituting the obtained value of the displacement parameter *C*_*PI*_ into the fourth term of the control Eq. (25) gives the value of the pull-in voltage *V*_*PI*_.

37 $$V_{PI} = \sqrt {\frac{{2\left[ {C \cdot \psi_{1} - C^{3} \cdot \psi_{2} + \psi_{4} \left( C \right)} \right] \cdot g^{3} \cdot \left( {EI} \right)^{ * } }}{{\psi_{3} \left( C \right) \cdot \varepsilon_{0} \cdot bl^{4} }}}$$

Taking the two-end-fixed nano-switch as an example, the instability occurs at the center of the movable electrode *W*(*X* = 0.5) when it is in the state of adhesion instability. Therefore, by substituting the obtained displacement undetermined parameter *C*_*PI*_ value under adhesion instability state and the first-order shape function in Table 1 into Eq. (28), the adhesion displacement* Y*_*PI*_ value of the two-end-fixed nano-switch can be obtained, that is,

38 $$Y_{PI} = C_{PI} \cdot \varphi \left( {0.5} \right)$$

The same applies to the remaining three types of boundary conditions.

## Result and discussion

In the numerical examples, we take the geometric and material parameters of the nano-switch structure as follows: length *l* = 1 μm, cross-sectional height *t* = 50 nm, width *b* = 5*t*, initial gap between the two electrode plates *g* = 50 nm, beam elastic modulus *E* = 76GPa, surface elastic modulus *E*^*s*^ = 1.22 Nm^−1^, and residual surface energy density *γ* = 0.89 Jm^−2^ [10].

**Fig. 3:**
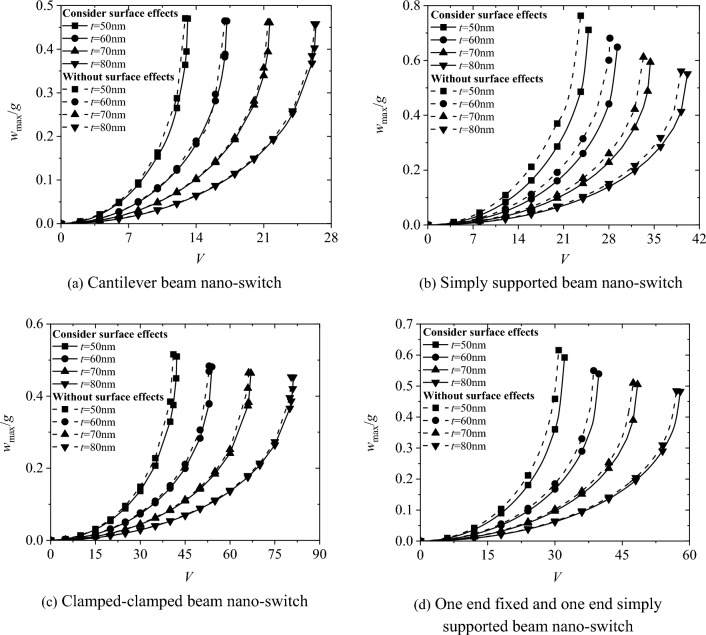
The influence of surface effect on the relationship between maximum displacement and voltage of nano-switches

Figure 3 shows the relationship between the maximum non-dimensional deflection wmax/g and the applied voltage for the four boundary conditions of the nano-switch. The results show that the maximum non-dimensional deflection *w*_*max*_*/g* increases continuously with the increase of the applied voltage. When the applied voltage reaches the critical pull-in voltage *V*_*PI*,_ the nano-switch undergoes pull-in instability and the movable electrode adheres to the fixed electrode. Surface effects will increase the pull-in voltage of the nano-switch. As the cross-sectional height t of the movable electrode increases from 50 to 80 nm with an increment of 10 nm, the pull-in voltage of the nano-switch increases and the influence of surface effects weakens.

**Fig. 4:**
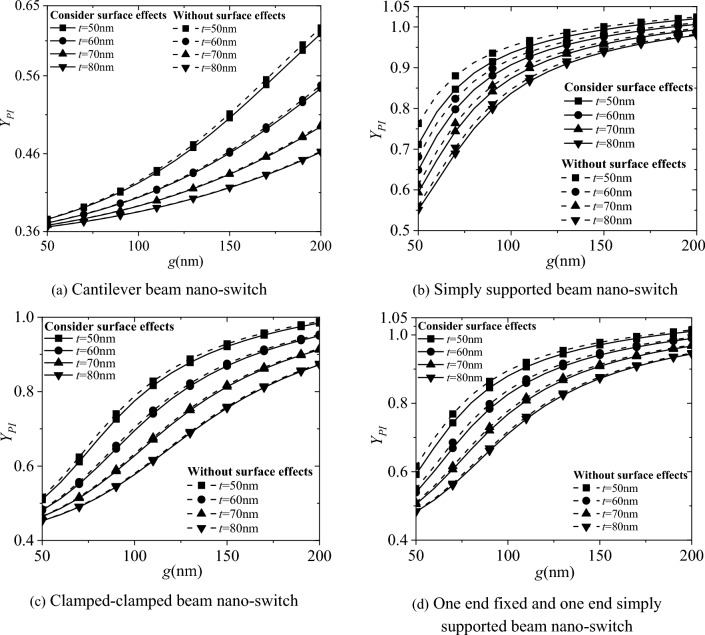
Effects of surface effect and electrode spacing g on the pull-in displacement *Y*_*PI*_ of nano-switches

Figure 4 shows the influence of surface effect and electrode spacing g on the pull-in displacement *Y*_*PI*_ of the four types of boundary conditions in the nano-switch. The results show that as the electrode spacing g increases, the pull-in displacement *Y*_*PI*_ of the nano-switch also increases. The pull-in displacement *Y*_*PI*_ of the nano-switch considering surface effect is smaller than that without considering the surface effect. Moreover, as the cross-sectional height t of the movable electrode increases by 10 nm from 50 to 80 nm, the pull-in displacement* Y*_*PI*_ of the nano-switch decreases continuously, and the influence of the surface effect gradually decreases as well.

**Fig. 5:**
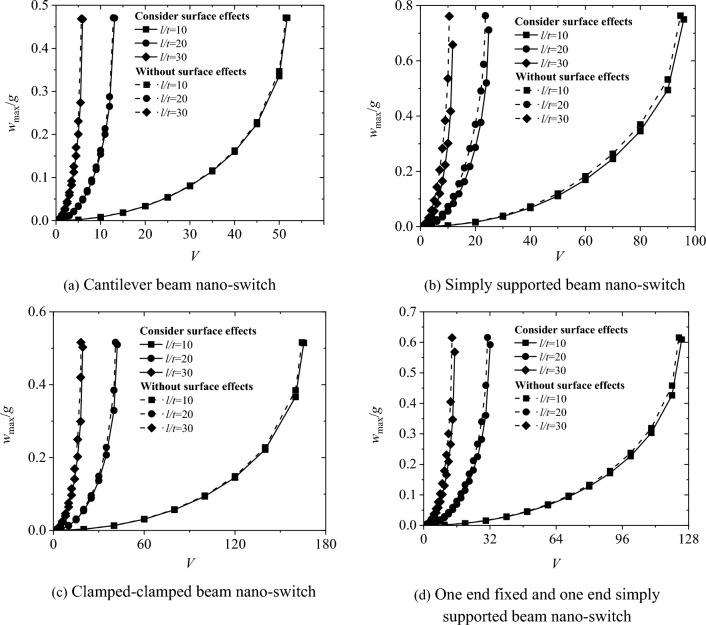
Effect of l/t on the pull-in characteristics of nano-switches

Figure 5 shows the influence of the length-to-height ratio l/t of the movable electrode on the switching characteristics of the nano-switch for the four boundary conditions. It can be seen from the graph that as the length-to-height ratio l/t of the movable electrode increases, the pull-in voltage *V*_*PI*_ of the nano-switch decreases. Similar to linear bending, this is because the increase in the length-to-height ratio l/t reduces the stiffness of the structure, making it more prone to deformation, and thus the pull-in voltage decreases.

**Fig. 6:**
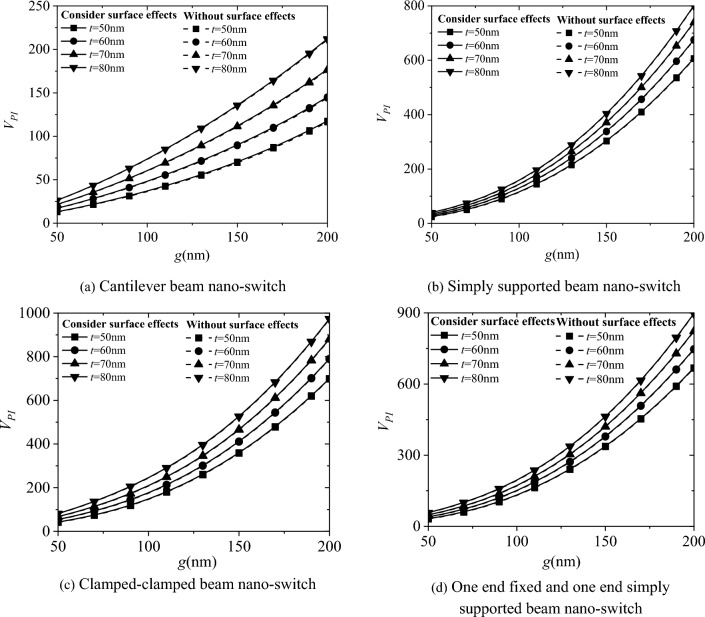
Effect of electrode spacing g on the pull-in voltage *V*_*PI*_ of nano-switches

Figure 6 shows the effect of electrode spacing g on the pull-in voltage *V*_*PI*_ of the four boundary conditions for the nano-switch. The results indicate that as the electrode spacing g increases, the pull-in voltage *V*_*PI*_ also increases. The pull-in voltage with and without considering surface effects are very close. Moreover, as the cross-sectional height t of the movable electrode increases from 50 to 80 nm in increments of 10 nm, the pull-in voltage of the nano-switch increases, consistent with the results obtained in Fig. 3.

**Fig. 7:**
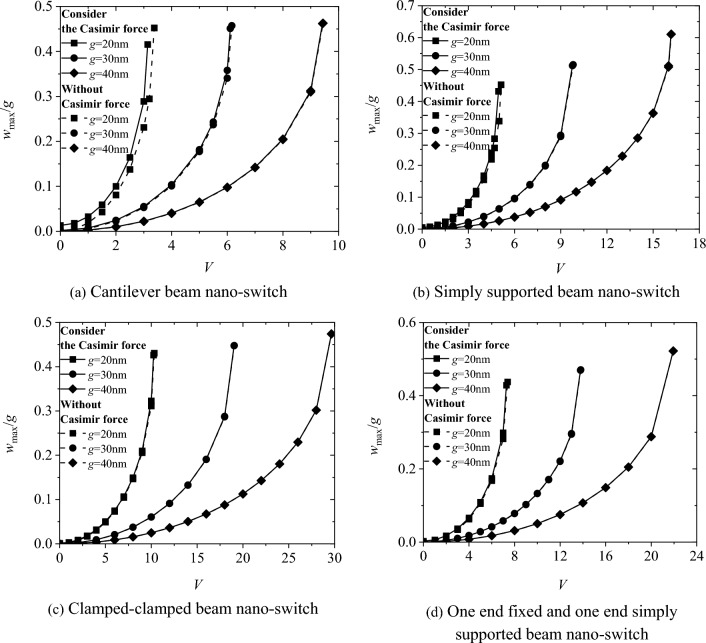
The effect of Casimir force on the pull-in characteristics of nano-switches

Figure 7 shows the effect of Casimir force on the switching characteristics of the four boundary conditions. The results indicate that the switching voltage *V*_*PI*_ considering the Casimir force is smaller than that without considering it, and the effect of Casimir force decreases as the electrode spacing g increases. At an external voltage V = 0, the maximum dimensionless deflection *w*_*max*_*/g* of the switch considering the Casimir force is not zero, indicating that the switch also undergoes deformation due to the influence of the Casimir force in the absence of an external electric field.

**Fig. 8:**
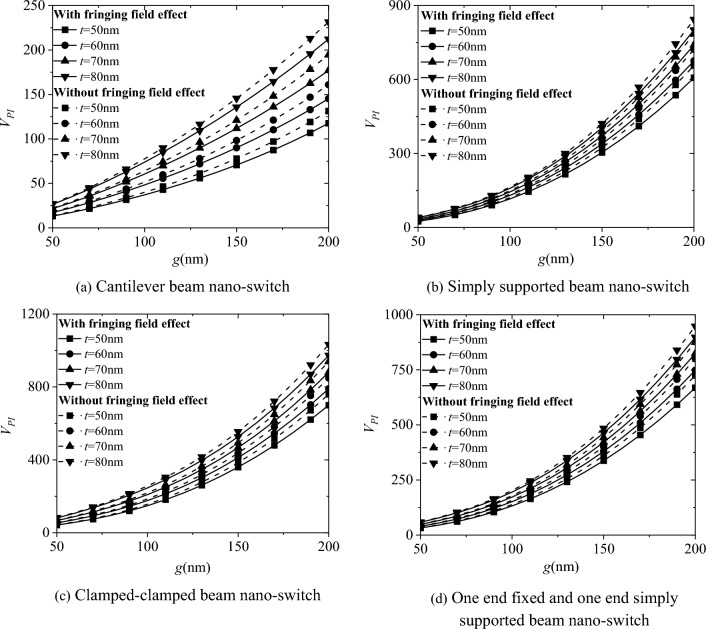
Effect of electric field edge effect on the pull-in voltage *V*_*PI*_ of nano-switches

Figure 8 shows the influence of the electric field edge effect on the pull-in voltage *V*_*PI*_ of four boundary conditions for the nano-switch. The results indicate that considering the electric field edge effect will reduce the nonlinear bending pull-in voltage *V*_*PI*_ of the nano-switch, and ignoring the effect of the electric field edge will over-predict the pull-in voltage *V*_*PI*_. As the electrode spacing g increases, the influence of the electric field edge effect also gradually increases.

**Fig. 9:**
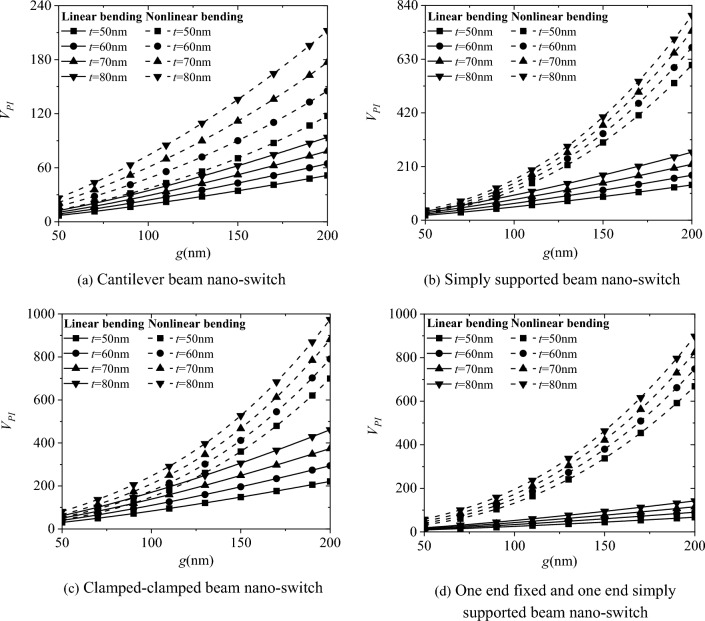
Comparative analysis of linear bending and nonlinear bending pull-in characteristics of nano-switches

Figure 9 shows a comparison of the pull-in characteristics between linear and nonlinear bending of the nano-switch. Comparing the results of linear and nonlinear bending, it can be concluded that the pull-in voltage *V*_*PI*_ of the nano-switch with nonlinear bending is higher than that of the one with linear bending, and ignoring the effect of geometric nonlinear deformation will lead to an underestimation of the pull-in voltage *V*_*PI*_ of the nano-switch. In addition, the influence of geometric nonlinear deformation on the pull-in voltage increases with the height t of the movable electrode cross-section, and also increases with the electrode spacing g.

**Fig. 10:**
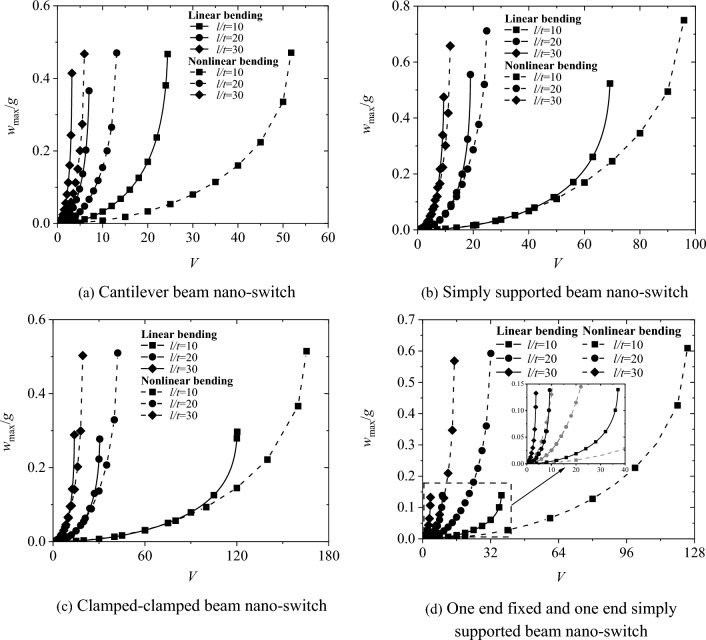
Comparative analysis of linear bending and nonlinear bending pull-in characteristics of nano-switches with different aspect ratios *l*/t

Figure 10 shows a comparison of the pull-in characteristics between linear and nonlinear bending of nano-switches with different length-to-height ratios. From the figure, it can be seen that the pull-in voltage *V*_*PI*_ of the nano-switch with nonlinear bending is also higher than that of the one with linear bending. Moreover, the influence of geometric nonlinear deformation on the pull-in voltage of the nano-switch decreases as the length-to-height ratio l/t of the movable electrode increases.

## Conclusion

This paper studies the influence of surface effects on the nonlinear bending and pull-in stability of nano-switches. Based on the Von Karman geometric nonlinear theory, the nonlinear bending control equation and boundary conditions of nano-switches considering surface effects are derived through the minimum potential energy principle, and the Galerkin method is used to solve the nonlinear bending control equation. The results show that surface effects increase the pull-in voltage and decrease the pull-in displacement of the nano-switch with nonlinear bending, and as the size of the nano-switch structure increases, the influence of surface effects decreases. Compared with the results of linear theory, the pull-in voltage of the nonlinear theory is larger. Geometric nonlinear effects increase with the size of the nano-switch structure and electrode spacing, but decrease with the increase of the length-to-height ratio of the nano-switch structure. This provides theoretical support and reference for the design and use of future nanodevices and nanomechanical systems.
